# Supplementary figures and images for: Clustering of fast gyrotactic particles in low-Reynolds-number flow
Source: PLoS One. 2022 Apr 7;17(4):e0266611. doi: 10.1371/journal.pone.0266611 (PMC8989315; doi:10.1371/journal.pone.0266611)

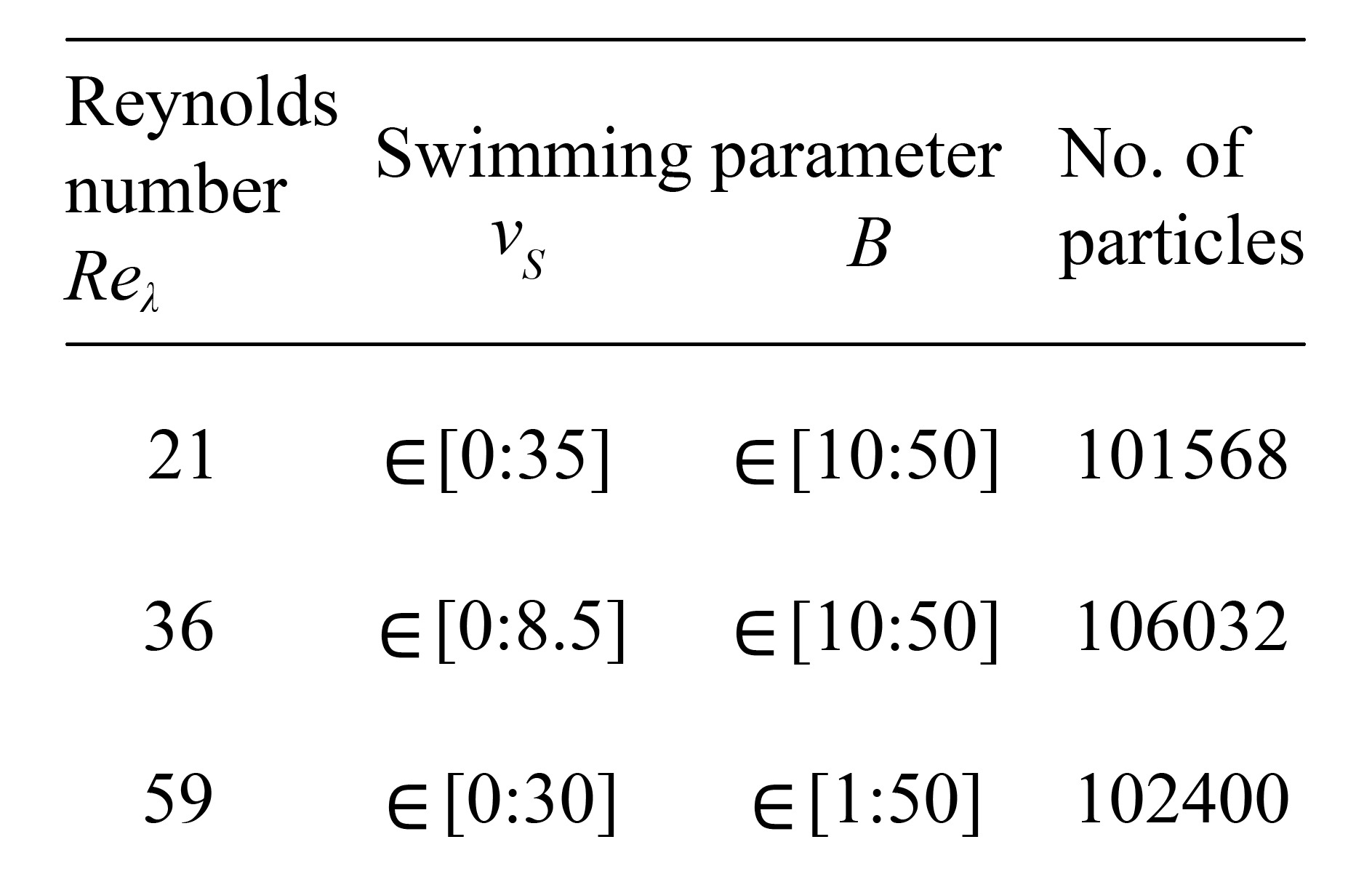

Supplement: S1 Table — Swimming parameters and number of particles used in each simulation for different values of Reynolds number. (TIF) [file pone.0266611.s001.tif]

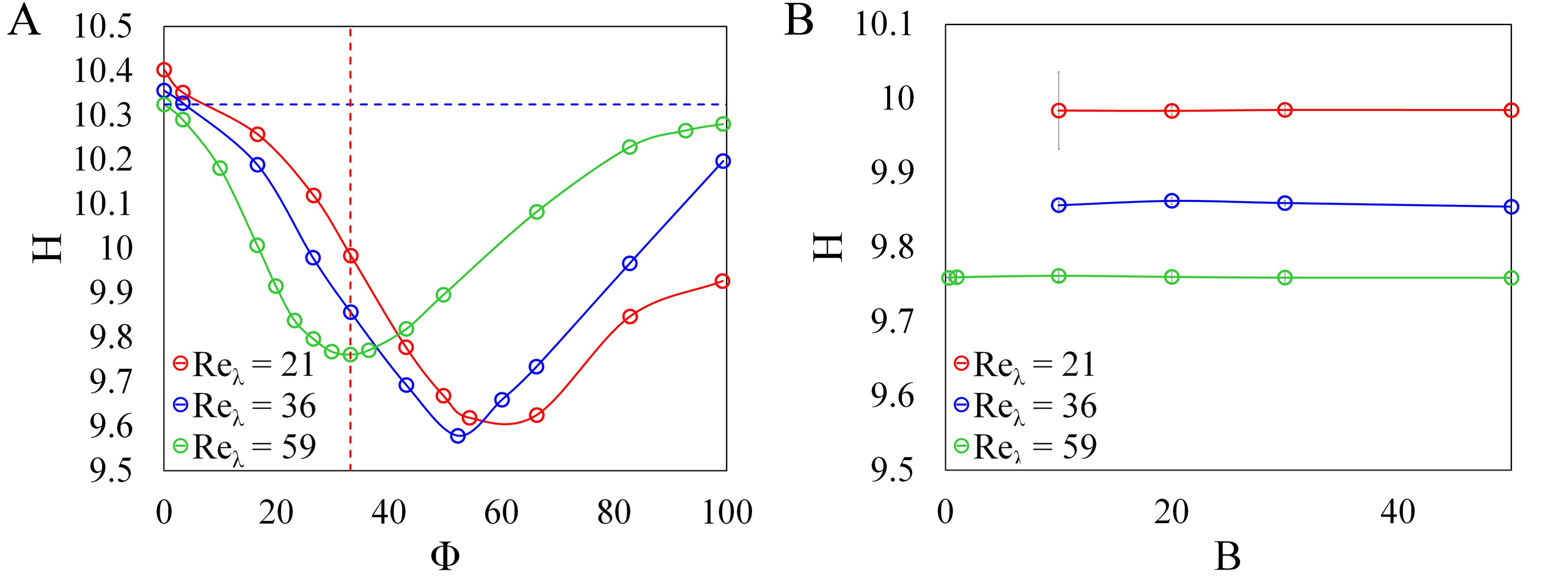

Supplement: S1 Fig — Changes in the entropy H with (A) swimming number Φ, and (B) reorientation time B for different values of Reynolds number Reλ at Φ = 33. (TIF) [file pone.0266611.s002.tif]

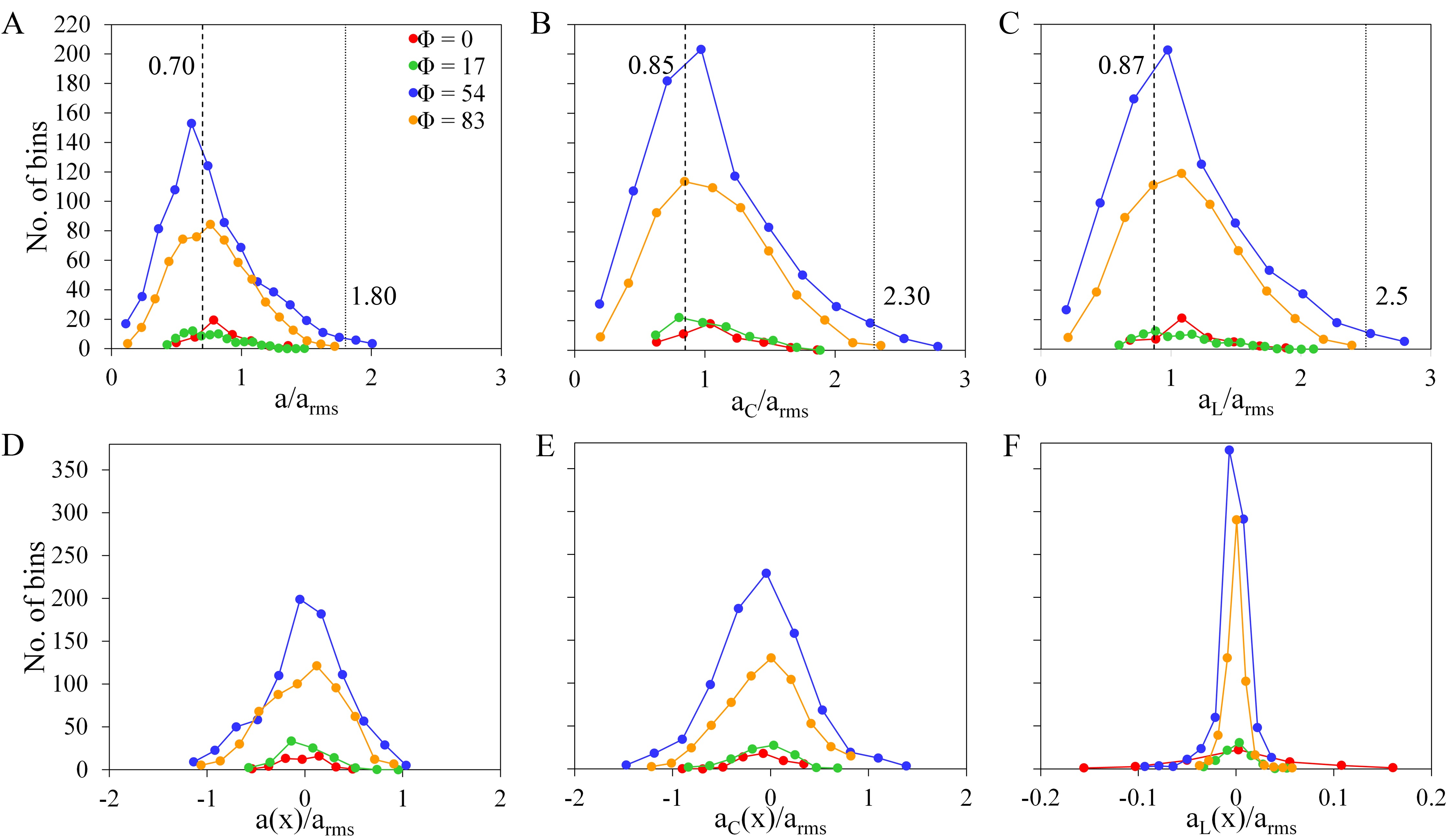

Supplement: S2 Fig — Histogram for (A) acceleration, (B) centripetal acceleration, and (C) longitudinal acceleration while (D-F) corresponds to the histograms for the x-components of the three accelerations. (TIF) [file pone.0266611.s003.tif]

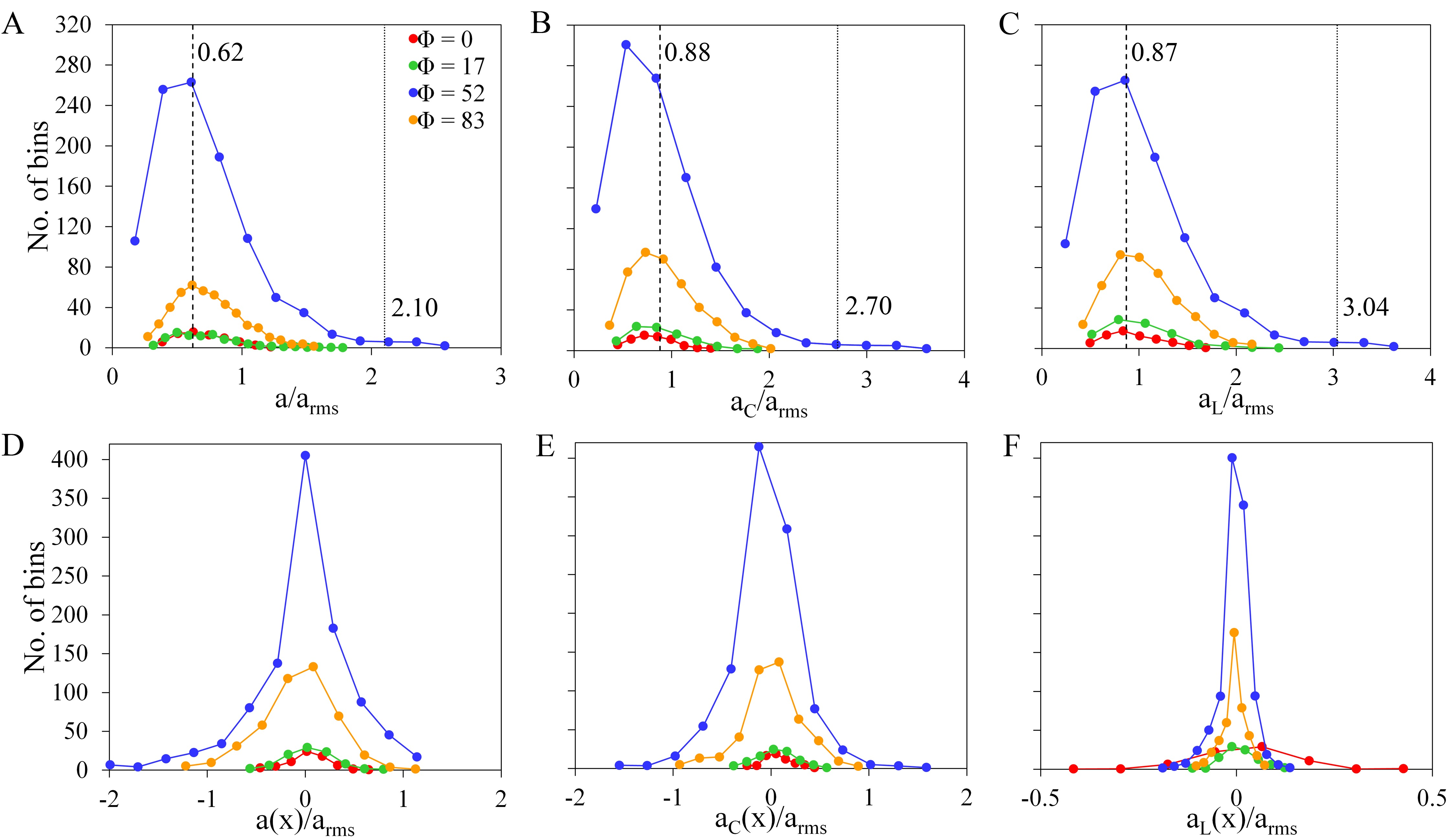

Supplement: S3 Fig — Histogram for (A) acceleration, (B) centripetal acceleration, and (C) longitudinal acceleration while (D-F) corresponds to the histograms for the x-components of the three accelerations. (TIF) [file pone.0266611.s004.tif]
