# Supplementary material for: Clustering of fast gyrotactic particles in low-Reynolds-number flow
Source: PLoS One. 2022 Apr 7;17(4):e0266611. doi: 10.1371/journal.pone.0266611 (PMC8989315; doi:10.1371/journal.pone.0266611)
Supplement: Attachment — Submitted filename: Response to Reviewers.docx [file pone.0266611.s005.docx]

**RESPONSE TO THE EDITOR**

**COMMENT 1:** Please ensure that your manuscript meets PLOS ONE's style requirements, including those for file naming. The PLOS ONE style templates can be found at

https://journals.plos.org/plosone/s/file?id=wjVg/PLOSOne_formatting_sample_main_body.pdf and

https://journals.plos.org/plosone/s/file?id=ba62/PLOSOne_formatting_sample_title_authors_affiliations.pdf.

**RESPONSE 1**: Thank you for the resources. We have

1. Added corresponding author’s initials beside email address.
2. Removed funding information from the Acknowledgements section.
3. Improved the citations of equations and figures in Methods and Results and discussions sections.

**COMMENT 2:** Thank you for stating the following in the Acknowledgments Section of your manuscript:

[We would like to thank the Department of Science and Technology (DOST)-SEI 252

Accelerated Science and Technology Human Resource Development Program (ASTHRDP) and University of San Carlos for supporting this research. M. Liponhay would also like to acknowledge the funding from DOST-CRADLE Program, Project No. 8419. Finally, we thank Professors Danilo M. Yanga, Christopher P. Monterola, and Christian M. Alis for the fruitful discussions about this work.]

We note that you have provided funding information that is not currently declared in your Funding Statement. However, funding information should not appear in the Acknowledgments section or other areas of your manuscript. We will only publish funding information present in the Funding Statement section of the online submission form.

Please remove any funding-related text from the manuscript and let us know how you would like to update your Funding Statement. Currently, your Funding Statement reads as follows:

[The author(s) received no specific funding for this work.]

Please include your amended statements within your cover letter; we will change the online submission form on your behalf.

**RESPONSE 2:** Concerning the Acknowledgments Section of the manuscript, we have removed the funding-related statement and incorporated this correction in the revised manuscript. We propose the following amendment to the funding statement:

*“JA and ML acknowledge the Department of Science and Technology (DOST)-SEI Accelerated Science and Technology Human Resource Development Program (ASTHRDP) and the University of San Carlos for supporting this research. ML acknowledges the Department of Science and Technology (DOST) of the Philippines with Project No. 8419, 2020 under the Collaborative R&D to Leverage the Economy (CRADLE) Program (https://s4cp.dost.gov.ph/programs/cradle/). The funders had no role in study design, data collection, and analysis, decision to publish, or preparation of the manuscript.”*

**COMMENT 3:** We note that you have stated that you will provide repository information for your data at acceptance. Should your manuscript be accepted for publication, we will hold it until you provide the relevant accession numbers or DOIs necessary to access your data. If you wish to make changes to your Data Availability statement, please describe these changes in your cover letter and we will update your Data Availability statement to reflect the information you provide.

**RESPONSE 3:** Thank you for the information. We are changing our Data Availability statement to

*“All preprocessed data files are available from 10.5281/zenodo.5904617, 10.5281/zenodo.5905360, and 10.5281/zenodo.5904923.”*

We have also mentioned this in our cover letter.

**RESPONSE TO THE REVIEWER**

Reviewer #1: This manuscript is on the clustering of fast gyro-tactic particles in low-Reynolds-number flow, which is beneficial to understand various ecological phenomena, for example, harmful algal blooms in lakes. The authors' efforts are appreciated, and the authors should consider the following points to improve further the quality of this manuscript.

**COMMENT 1:** The primary method used in this paper was reported in previous work by Durham et al. (Durham WM, Climent E, Barry M, De Lillo F, Boffetta G, Cencini M, et al. Turbulence drives microscale patches of motile phytoplankton. Nature communications. 2013). This work presents swimming particles' velocity and acceleration characteristics for different parameters. However, the numerical analysis in this work has not been verified by some experiments and relevant literature. At least, more cases of numerical simulation should be performed and analyzed for flows of various Reynolds numbers to make the conclusion powerful.

**RESPONSE 1:** We thank the reviewer for this comment. We have performed additional numerical simulations using different Reynolds numbers, where clustering of gyrotatic cells is observed inside the vortices in the previous studies. We use similar values to confirm whether the mechanism of particle clustering discussed in our study can be observed in such flows.

We found the same behavior for $Re_{\lambda}=[21, 36]$ – the presence of clustering with the same characteristics with respect to velocity and acceleration distribution as when $Re_{\lambda}=59$, which supports our conclusions. Shown below are the corresponding snapshots of the distribution of particles for $Re_{\lambda}=[21, 36]$ for different swimming numbers.

We have also provided additional discussions in the Results and discussions section (in the third paragraph of the Cluster formation subsection and in the last paragraph of the Velocity and acceleration characteristics of swimming particles subsection) to support our conclusions and have incorporated the results in the manuscript as supporting information.

**COMMENT 2:** The conclusion is given for fast gyrotactic particles in low-Reynolds-number flow. More limitations are required to specify the scope of the conclusion, for example, the range of low-Reynolds-number flow.

**RESPONSE 2:** We thank the reviewer for the suggestion. We agree with the reviewer, and we have included the range of low-Reynolds-number flow and the basis of selection of these values in Paragraph 2 of the Numerical procedure subsection of Methods.

Our findings that motile particles cluster outside vortices when also happen for low Re numbers, i.e., when the particle speed is high enough. Given these observations, our data and findings now better support the conclusions with its scope for fluid flow at low Reynolds number and high particle speed. The results of the numerical experiments using these values have been discussed in the Results and Discussions and are presented in the Supporting information section.

In addition to the revisions made to the manuscript above, we have also made corrections in the values used to de-dimensionalize the swimming parameters, changing the values of the swimming parameters. These changes only affect the swimming parameter values reported in the figures (axis values in Figs 3 and 4 and legends of Figs 2-7) and do not affect the trend in our plots or the analyses of the data. We have incorporated these corrections in the Methodology and Results and Discussions sections of the manuscript.

We also added a new paragraph at the beginning of the Results and discussion section to add clarity in the organization of the presentation of results.
