# Supplementary material for: Clustering of fast gyrotactic particles in low-Reynolds-number flow
Source: PLoS One. 2022 Apr 7;17(4):e0266611. doi: 10.1371/journal.pone.0266611 (PMC8989315; doi:10.1371/journal.pone.0266611)
Supplement: Attachment — Submitted filename: Response to Reviewers.docx [file pone.0266611.s006.docx]

**RESPONSE TO THE EDITOR**

Journal Requirements:

Please review your reference list to ensure that it is complete and correct. If you have cited papers that have been retracted, please include the rationale for doing so in the manuscript text, or remove these references and replace them with relevant current references. Any changes to the reference list should be mentioned in the rebuttal letter that accompanies your revised manuscript. If you need to cite a retracted article, indicate the article’s retracted status in the References list and also include a citation and full reference for the retraction notice.

Response:

We thank the editor for this comment. We have checked our reference list and found no articles that have been retracted. We made no major changes in the reference list except for a few additional information added in reference numbers 8, 21, 24, and 25.

**RESPONSE TO THE REVIEWER**

Reviewer #1:

(1) Please check Eq.2; it is not written correctly. p->dp/dt

Thank you for spotting this error. We have made corrections to the expression of Eq. 2 and have incorporated it in the revised manuscript.

(2) The references for Eqs. 1 and 2 should be given.

We thank the reviewer for this comment. We have now cited the reference for Eqs. 1 and 2 in line number 66. We have also checked if there are parts in the manuscript where we may have failed to properly cite the references and made the necessary corrections in the revised manuscript. We cited the references in line numbers 29-30 and line numbers 120-122.

(3) Figures are unclear, and authors should make sure all figures are clear enough for reading.

Thank you for pointing out this concern. We have improved our figures and increased their resolutions making sure that they are all clear enough for reading. We have also revised Fig 7, S3 Fig and S4 Fig so that the axis titles are shown.

(4) Texts and languages should be improved further. For example, Navier-Stokes NS equation -> Navier-Stokes equation.

Regarding this concern, we have now improved the texts and languages used in the manuscript.

We have changed the Navier-Stokes NS equation to Navier-Stokes (NS) equation, as NS equation is repeatedly used in the manuscript. Similar necessary changes have also been made throughout the manuscript to remove inconsistencies in the usage of other terms and abbreviations.

We have also significantly improved the clarity of the discussions in our manuscript by revisiting our choices of words and by polishing our use of the English language.
